# Industry Payments to Physician Specialists Who Prescribe Repository Corticotropin

**DOI:** 10.1001/jamanetworkopen.2018.0482

**Published:** 2018-06-29

**Authors:** Daniel M. Hartung, Kirbee Johnston, David M. Cohen, Thuan Nguyen, Atul Deodhar, Dennis N. Bourdette

**Affiliations:** 1 College of Pharmacy, Oregon State University, Corvallis; 2 Oregon Health & Science University, Portland; 3 Division of Nephrology & Hypertension, Department of Medicine, Oregon Health & Science University, Portland; 4 School of Public Health, Oregon Health & Science University, Portland; 5 Division of Arthritis & Rheumatic Diseases, Department of Medicine, Oregon Health & Science University, Portland; 6 Department of Neurology, Oregon Health & Science University, Portland

## Abstract

**Importance:**

Despite great expense and little evidence supporting use over corticosteroids, prescriptions for repository corticotropin (H. P. Acthar Gel; Mallinckrodt Pharmaceuticals) have increased markedly. Aggressive sales tactics and payments from the manufacturer may influence prescribing behavior for this expensive medication.

**Objective:**

To characterize industry payments to physician specialists who prescribe corticotropin in the Medicare program.

**Design, Setting, and Participants:**

This study was a cross-sectional analysis of Centers for Medicare & Medicaid Services 2015 Part D prescribing data linked to 2015 Open Payments data. Nephrologists, neurologists, and rheumatologists with more than 10 corticotropin prescriptions (frequent prescribers) in 2015 were included.

**Exposures:**

Frequency, category, and magnitude of corticotropin-related payments from Mallinckrodt recorded in the Open Payments database.

**Main Outcomes and Measures:**

Frequency, category, and magnitude of corticotropin-related payments from Mallinckrodt, as well as corticotropin prescriptions and expenditures for Medicare beneficiaries.

**Results:**

Of the 235 included physicians, 65 were nephrologists; 59, neurologists; and 111, rheumatologists. A majority of frequent corticotropin prescribers (207 [88%]) received corticotropin-related payments from Mallinckrodt. The median (range) total payment for 2015 was $189 ($11-$138 321), with the highest payments ranging from $56 549 to $138 321 across the specialties. More than 20% of frequent prescribers received more than $10 000 and the top quartile of recipients received a median (range) of $33 190 ($9934-$138 321) in total payments per prescriber. Payments for compensation for services other than consulting contributed the most to the total amount. Mallinckrodt payments were positively associated with greater Medicare spending on corticotropin (β = 1.079; 95% CI, 1.044-1.115; *P* < .001), with every $10 000 in payments associated with a 7.9% increase (approximately $53 000) in Medicare spending on corticotropin. There was no association between corticotropin-related payments and spending on prescriptions for synthetic corticosteroids.

**Conclusions and Relevance:**

In this study, most nephrologists, neurologists, and rheumatologists who frequently prescribe corticotropin received corticotropin-related payments from Mallinckrodt. These findings suggest that financial conflicts of interest may be driving use of corticotropin in the Medicare program.

## Key Points

**Question:**

What is the association of industry payments to physicians and prescriptions for repository corticotropin (H. P. Acthar Gel; Mallinckrodt Pharmaceuticals)?

**Findings:**

In this cross-sectional study of 235 specialist physicians who frequently prescribe corticotropin to Medicare beneficiaries, 207 (88%) received a monetary payment from the drug’s maker, with more than 20% of frequent prescribers receiving more than $10 000. There was a significant association between higher dollar amounts paid to these prescribers and greater Medicare spending on their corticotropin prescriptions.

**Meaning:**

Financial conflicts of interest among physicians may be driving corticotropin expenditures for the Medicare program.

## Teaser

This study uses Medicare Part D and Open Payments data to characterize payments to physicians from the manufacturer of repository corticotropin gel (H. P. Acthar Gel, an ACTH analogue) and associations between those payments and the number of prescriptions and Medicare expenditures for the drug in 2015.

## Introduction

It is well documented that the pharmaceutical industry spends a substantial amount of money to influence the decisions of physicians who use their products.^1,2^ In 2016, pharmaceutical, biotechnology, and device manufacturers paid 631 000 physicians more than $2 billion in food and beverages, gifts, educational materials, and speaker and consulting services.^3^ Although physicians often deny these payments affect their prescribing decisions, the evidence suggests the contrary.^4,5,6,7,8,9,10,11^ Studies consistently demonstrate pharmaceutical industry payments are associated with more prescriptions, greater prescription costs, and higher branded drug prescribing.^5,9,11,12^ Furthermore, several well-designed studies have demonstrated association specificity and a response gradient (higher payments associated with greater prescribing), suggesting the association is causal.^5,7,10^

As high-priced therapies become the norm for many conditions, it is imperative that prescribing decisions are evidence based and free from undue commercial influence. Expensive therapies with uncertain or insufficient evidence supporting their use should be particularly scrutinized. A prime example of such a treatment is repository corticotropin (H. P. Acthar Gel; Mallinckrodt Pharmaceuticals). Originally approved by the US Food and Drug Administration (FDA) in 1952, corticotropin has received considerable attention in recent years because of the dramatic increase in its cost. Corticotropin is a porcine-derived biologic preparation with a proprietary manufacturing process and for decades was available for less than $50 per vial. In 2007, Questcor Pharmaceuticals, who acquired the license for corticotropin in 2001 for $100 000, raised the acquisition price for a 5-mL vial from $1650 to $23 269 (a 14-fold increase).^13^ Mallinckrodt Pharmaceuticals, which acquired Questcor in 2014, has continued to raise the price to its current acquisition cost of $38 892.^14^

There is a lack of evidence supporting the use of corticotropin for most indications.^15^ Corticotropin was approved prior to the 1962 Kefauver-Harris Amendment to the US Food, Drug, and Cosmetics Act, which added the requirement of evidence of efficacy to the FDA approval process.^16^ As a consequence, corticotropin’s original label lacked evidence derived from controlled clinical trials that meet current standards for FDA approval for any indication. Over the ensuing years, corticotropin’s label has been revised several times, first as part of the FDA’s Drug Efficacy Study Implementation review in 1971, and then with the addition of indications for the treatment of relapses of multiple sclerosis in 1979 and infantile spasms in 2010.^17^ The only randomized clinical trials of corticotropin to treat relapses of multiple sclerosis show it to be more effective than placebo (n = 197) but no better than methylprednisolone (n = 61).^18,19^ The use of corticotropin for infantile spasms is supported by 5 controlled clinical trials of between 24 and 50 (mean, 34) participants.^20,21,22,23,24^ Corticotropin is also indicated and promoted on its website for systemic lupus erythematosus, proteinuria in nephrotic syndrome, dermatomyositis and polymyositis, rheumatoid arthritis, symptoms of sarcoidosis, and inflammatory conditions of the eye, such as uveitis. With the exception of a small placebo-controlled trial of 38 individuals with systemic lupus erythematosus demonstrating equivocal efficacy compared with placebo,^25^ the clinical evidence supporting the efficacy of corticotropin for these indications generally consists of small (n < 25) uncontrolled trials and case reports.^26^

Because of its very high price, corticotropin is a major expenditure for public insurance programs in the United States. From 2011 to 2015, spending on corticotropin in the US Medicare program increased 10-fold, totaling more than $1 billion during this period.^27^ In 2015 alone, Medicare spent more than $500 million on corticotropin, making it one of the most expensive drugs paid for by the program.^28^ The continued growth in corticotropin use is peculiar given its very high cost, widespread negative media coverage, and notable lack of evidence supporting its use over lower-cost synthetic corticosteroids.^29,30^ Our experience suggests aggressive marketing of the drug partly accounts for increasing use. While media reports also indicate that industry payments to corticotropin prescribers may have a role, the prevalence, magnitude, and effect of corticotropin-related payments have not been systematically described.^30^ The goal of this research was to characterize payments to physicians who frequently prescribe corticotropin to Medicare beneficiaries and determine whether payment amounts are positively correlated with prescribing intensity.

## Methods

### Study Sample and Data Sources

Using several publicly accessible databases provided by the Centers for Medicare & Medicaid Services (CMS), we conducted a cross-sectional study to describe pharmaceutical industry payments to nephrologists, neurologists, and rheumatologists who frequently prescribe corticotropin in the Medicare Part D program. To identify Medicare prescribers of corticotropin, we used Medicare Part D Public Use Files (PUFs) from 2015.^31^ Medicare Part D PUFs contain prescription information aggregated at different levels for drugs paid for by Medicare Part D stand-alone plans and Medicare Advantage plans and include data for 71% of all 56 million Medicare beneficiaries.^28^ The Detailed Prescriber PUF summarizes total prescriptions and costs for every drug with more than 10 prescriptions by a prescriber. We used the Detailed Prescriber PUF to identify and determine the specialty of physicians who prescribed corticotropin in 2015. Drugs with 10 or fewer prescriptions per prescriber are suppressed by CMS to protect patient confidentiality. We focused on rheumatology, neurology, and nephrology specialists, who are the largest prescribers of corticotropin.^27^ Because this study used publicly available data, the Oregon Health & Science University institutional review board deemed it exempt from review. Strengthening the Reporting of Observational Studies in Epidemiology (STROBE) reporting guidelines were followed.

To summarize characteristics of these frequent corticotropin prescribers, we used the Medicare Part D Prescriber PUF and data from CMS Physician Compare. The Part D Prescriber PUF contains drug utilization data (eg, total prescriptions, beneficiaries, and expenditures for Medicare) for all participating Part D prescribers. We used the CMS Physician Compare data set to characterize other practice-related data, such as year of graduation from medical school and practice size. The CMS Physician Compare data set contains general demographic, training, and practice site information for all health care professionals who provide Medicare services. For comparison, we also summarized Part D prescribing and practice characteristics for all other neurologists, rheumatologists, and nephrologists who provide Medicare services.

To obtain information on industry payments, we used 2015 calendar year files from the Open Payments website. The Open Payments program began collecting information about payments from drug and device companies to physicians and teaching hospitals in 2013.^32^ Reported payments include consulting fees, honoraria, gifts, entertainment, food and beverage, travel, and research payments and ownership interests.^33^ All payments with a cash value of at least $10, or $100 in aggregate in 1 calendar year, must be reported. The payment data also report the specific product (eg, drugs, devices, biologics) to which the payment was related.

For corticotropin prescribers identified in the Medicare Part D PUF, we manually reviewed his or her Open Payments physician demographic characteristics record to verify concordance. While PUFs are indexed by each prescriber’s National Prescriber Identifier, Open Payments only includes name and location as identifiers. If prescriber specialty differed between the Medicare Part D PUF and Open Payments data, we used CMS Physician Compare data to verify the physician’s specialty. If a prescriber was not found within Open Payments, we verified their specialty in Physician Compare and considered them to not have received any industry payments. Only 1 prescriber’s specialty designation was misclassified in the Medicare PUF.

After we verified the linkage between each corticotropin prescriber and his or her Open Payments record, we extracted information from the Open Payments data on total payment amounts, number of transactions, and types of payments from Mallinckrodt Pharmaceuticals in 2015. We only included Mallinckrodt payments if the payment indicated it was for corticotropin. Our analysis included general payments (eg, honoraria or consulting), research payments, and ownership payments. We also extracted and summarized payments from Mallinckrodt unrelated to corticotropin and all other companies.

### Statistical Analysis

We analyzed the prevalence, frequency, category, and monetary amount of corticotropin-related payments by Mallinckrodt. Physician and payment characteristics were summarized with descriptive statistics as appropriate depending on their nature and distribution.

We evaluated the association between payment amounts and Medicare expenditures on corticotropin in 2 ways. First, we grouped prescribers into categories by the total dollar amounts of corticotropin-related payments received from Mallinckrodt and examined the trend in total corticotropin prescriptions and expenditures using the Kendall rank correlation coefficient statistic (Kendall τ). We also conducted multivariable linear regression analyses to evaluate whether payment amounts were positively correlated with corticotropin Medicare expenditures. Our dependent variable was the dollar amount that Medicare spent on corticotropin. The primary independent variable was the total Mallinckrodt corticotropin-related payment to these specialists. Similar to DeJong et al,^7^ we controlled for the following covariates: physician specialty, sex, group practice size, years since graduation from medical school, and geographic region. To adjust for differences in other prescribing behaviors, we included covariates measuring the number of noncorticotropin prescriptions per beneficiary and the cost of noncorticotropin prescriptions per claim. We also adjusted for total payments unrelated to corticotropin from industry (eg, payments from other companies) to control for differences in engagement with other companies. For our primary analysis, we transformed the dependent variable with natural logarithmic transformation because Medicare spending data were right skewed. Relative to an untransformed model, using a logarithmic-transformed dependent variable resulted in improved model fit and diagnostic performance.

Finally, to assess the specificity of the association between corticotropin-related payments and corticotropin spending, we conducted a falsification test to evaluate the association between corticotropin-related payments and synthetic corticosteroid spending. Using the same modeling approach, we tested the association between corticotropin-related payments and spending for prednisone, methylprednisolone, prednisolone, dexamethasone, and cortisone. A positive relationship between corticotropin-related payments and corticosteroid spending would suggest any observed association between corticotropin-related payments and corticotropin spending may be confounded by other factors, such as patient severity of illness. For prescribers with no recorded corticosteroid spending, we imputed 0.001 before performing log transformation.

Analyses were conducted using SAS statistical software, version 9.4 (SAS Institute), and R statistical software, version 3.3.3 (R Foundation for Statistical Computing). All tests were 2-sided and *P* values less than .05 were considered statistically significant.

## Results

In 2015, Medicare spent $504 million for 11 209 corticotropin prescriptions written by 1743 prescribers. More than half of this expenditure ($266 197 661) was attributable to 300 prescribers (17.2%) with more than 10 corticotropin prescriptions. Most of these prescribers (n = 235) were identified, and verified, as rheumatologists (n = 111), neurologists (n = 59), or nephrologists (n = 65). Of the remaining 65 frequent prescribers, 24 were listed as internists, 19 were pulmonologists, 8 had other specialty designations (1 each for allergy and immunology, dermatology, diagnostic radiology, emergency medicine, family practice, neurosurgery, ophthalmology, and pain management), and 14 were other midlevel practitioners (9 nurse practitioners and 5 physician assistants).

The characteristics of the 235 frequent corticotropin prescribers and all other Medicare prescribers in their specialty groups (n = 26 046) are summarized in Table 1. In 2015, Medicare spent a total of $203 255 335 for corticotropin prescribed by these 235 physicians (median [interquartile range], $678 706 [$465 974-$984 020] per prescriber). Expenditures for corticotropin among frequent prescribers accounted for 37% of their total Part D expenditures, but ranged from 21% for neurologists to 54% for nephrologists. Frequent corticotropin prescribers were more likely to practice in smaller practices, write more prescriptions, and prescribe more expensive drugs than their specialty peers. Frequent corticotropin prescribers also prescribed more synthetic corticosteroids than their peers. Of these 235 physician prescribers of corticotropin, 25 (11%) practiced within an academic medical center.

**Table 1.** Characteristics of Frequent Prescribers of Repository Corticotropin in the Medicare Program in 2015

Characteristic	Nephrology		Neurology		Rheumatology		All 3 Specialties	
	Frequent Corticotropin Prescribers (n = 65)	Other Prescribers (n = 8213)	Frequent Corticotropin Prescribers (n = 59)	Other Prescribers (n = 13 325)	Frequent Corticotropin Prescribers (n = 111)	Other Prescribers (n = 4508)	Frequent Corticotropin Prescribers (n = 235)	Other Prescribers (n = 26 046)
Male, No. (%)	50 (76.9)	6085 (74.1)	41 (69.5)	9305 (69.8)	74 (66.7)	2626 (58.3)	165 (70.2)	18 016 (69.2)
Group practice size, No. (%)								
1	12 (18.5)	1068 (13.0)	22 (37.3)	2647 (19.9)	33 (29.7)	965 (21.4)	67 (28.5)	4680 (18.0)
2-10	22 (33.8)	1999 (24.3)	9 (15.3)	1509 (11.3)	35 (31.5)	735 (16.3)	66 (28.1)	4243 (16.3)
11-50	18 (27.7)	1747 (21.3)	8 (13.6)	1252 (9.4)	10 (9.0)	389 (8.6)	36 (15.3)	3388 (13.0)
>50	13 (20.0)	3399 (41.4)	20 (33.9)	7917 (59.4)	33 (29.7)	2419 (53.7)	66 (28.1)	13 735 (52.7)
Time since graduation, mean (SD), y^a^	23.5 (10.5)	22.9 (11.2)	26.6 (9.4)	23.4 (12.1)	26.6 (10.5)	24.4 (12.1)	25.7 (10.3)	23.4 (11.8)
US geographic region, No. (%)								
Northeast	13 (20.0)	1720 (20.9)	9 (15.3)	3267 (24.5)	25 (22.5)	1083 (24.0)	47 (20.0)	6070 (23.3)
Midwest	10 (15.4)	1723 (21.0)	12 (20.3)	2885 (21.7)	24 (21.6)	956 (21.2)	46 (19.6)	5564 (21.4)
South^b^	33 (50.8)	3118 (38.0)	24 (40.7)	4478 (33.6)	42 (37.8)	1563 (34.7)	99 (42.1)	9159 (35.2)
Pacific West	7 (10.8)	1117 (13.6)	8 (13.6)	1896 (14.2)	14 (12.6)	658 (14.6)	29 (12.3)	3671 (14.1)
Mountain West	2 (3.1)	535 (6.5)	6 (10.2)	799 (6.0)	6 (5.4)	248 (5.5)	14 (6.0)	1582 (6.1)
Corticotropin prescribing								
Total corticotropin expenditure, $	41 665 749	NR	41 056 933	NR	120 532 653	NR	203 255 335	NR
Corticotropin expenditure per prescriber, median (IQR), $	506 718 (420 884-659 918)	NR	614 760 (443 558-737 420)	NR	824 545 (591 100-1 172 163)	NR	678 706 (465 974-984 020)	NR
Corticosteroid prescribing^c^								
Count of frequent prescribers, No. (%)	49 (75.4)	4375 (53.2)	37 (62.7)	3890 (29.2)	111 (100)	4378 (97.1)	197 (83.8)	12 643 (48.5)
Total corticosteroid expenditure, $	24 782	1 802 668	62 551	1 774 892	1 145 859	23 071 018	1 233 192	26 648 578
Corticosteroid expenditure per prescriber, median (IQR), $	273 (146-273)	241 (140-440)	825 (313-1856)	279 (161-516)	6043 (3210-6043)	3607 (1660-6470)	2444 (460-6899)	450 (199-2113)
All medication prescribing								
Total expenditure, $	75 789 407	2 531 033 434	197 296 370	6 974 756 737	275 778 740	3 444 695 122	548 864 517	12 950 485 292
Expenditure per prescriber, median (IQR), $	1 035 563 (702 181-1 335 092)	220 798 (84 500-412 292)	3 000 411 (2 002 159-3 990 935)	245 589 (43 037-662 864)	2 299 877 (1 556 368-3 070 141)	584 817 (252 536-1 037 950)	1 932 495 (1 168 339-3 008 416)	275 396 (77 177-639 819)
Prescriptions per prescriber, median (IQR), No.	2373 (1604-3607)	1432 (642-2532)	3803 (2152-6630)	999 (278-2266)	3801 (2520-5501)	1967 (910-3593)	3377 (2105-5501)	1301 (451-2584)
Expenditure per prescription, median (IQR), $	380 (305-530)	144 (93-213)	677 (450-1322)	210 (113-358)	604 (436-758)	285 (196-396)	530 (378-823)	195 (113-321)

Abbreviations: IQR, interquartile range; NR, not reported.

^a^ Graduation year missing for 4.3% of corticotropin prescribers and 6.2% of other prescribers.

^b^ One physician from Puerto Rico was categorized into South.

^c^ Corticosteroids include prednisone, methylprednisolone, prednisolone, dexamethasone, and cortisone.

In 2015, Mallinckrodt paid $11 442 866 ($7 325 957 in general payments and $4 116 908 in research payments) to 10 491 physicians for corticotropin (10 452 for general payments and 97 for research payments). Among frequent corticotropin prescribers in our study, Mallinckrodt made a total of 5315 corticotropin-related payments to 207 prescribers (88% of frequent corticotropin specialist prescribers), totaling $2 213 727 ($1 986 926 in general payments and $226 801 in research payments). Table 2 summarizes the distribution and types of corticotropin-related payments made by Mallinckrodt to these frequent prescribers. Most nephrologists and nearly all neurologists and rheumatologists received at least 1 corticotropin-related payment from Mallinckrodt. Overall, while the median (range) total payment amounts during the year were modest at $189 ($11-$138 321), maximum total payment amounts were as high as $56 549 for nephrology, $120 387 for neurology, and $138 321 for rheumatology. The median (range) total payment for the top quartile of the 207 prescribers who received at least 1 payment was $33 190 ($9934-$138 321) overall, but ranged from $5249 for nephrologists to $41 405 for neurologists. Neurologists were the most likely to receive a payment (93.2% vs 78.5% for nephrologists and 91.0% for rheumatologists), had the greatest number of transactions (20 vs 5 for nephrologists and 13 for rheumatologists), and received the highest median total dollar amount over the year ($476 vs $118 for nephrologists and $207 for rheumatologists). Differences in total payments were driven by a higher likelihood of consulting, travel, and compensation for services other than consulting. Payments to neurologists for services other than consulting contributed the most to this difference (median [range] total payment amount, $15 865 [$1990-$91 400]). Research-related payments totaled $226 801 for 12 prescribers (5.1%). They were particularly high for 4 rheumatologists, who received a total of $214 281 (median [range], $53 208 [$32 865-$75 000]) during the year. No prescribers received ownership-related payments. As shown in eTable 1 in the Supplement, payments to these prescribers unrelated to corticotropin were also common and substantial.

**Table 2.** Repository Corticotropin–Related Payments to Frequent Physician Prescribers of Corticotropin From Mallinckrodt in 2015

Payments	Nephrology (n = 65)	Neurology (n = 59)	Rheumatology (n = 111)	Total (n = 235)
Any payment type				
Prescribers, No. (%)	51 (78.5)	55 (93.2)	101 (91.0)	207 (88.1)
Total payments, $	180 621	878 069	1 155 038	2 213 727
Total payments per prescriber, median (range), $	118 (12-56 549)	476 (11-120 387)	207 (12-138 321)	189 (11-138 321)
Total transactions, No.	517	2219	2579	5315
Transactions per prescriber, median (range), No.	5 (1-86)	20 (1-236)	13 (1-199)	11 (1-236)
Payment per transaction, median (range), $	22 (12-767)	28 (6-722)	22 (5-3671)	23 (5-3671)
Specific payment category				
Compensation for services other than consulting				
Prescribers, No. (%)	7 (10.8)	24 (40.7)	32 (28.8)	63 (26.8)
Total payments per prescriber, median (range), $	12 900 (2200-37 450)	15 865 (1990-91 400)	14 100 (650-70 650)	14 700 (650-91 400)
Travel				
Prescribers, No. (%)	6 (9.2)	21 (35.6)	33 (29.7)	60 (25.5)
Total payments per prescriber, median (range), $	5579 (62-14 690)	4253 (183-29 508)	2034 (10-28 061)	3213 (10-29 508)
Consulting				
Prescribers, No. (%)	4 (6.2)	20 (33.9)	26 (23.4)	50 (21.3)
Total payments per prescriber, median (range), $	2863 (1500-7500)	2700 (400-7400)	2680 (2500-15 860)	2700 (400-15 860)
Research				
Prescribers, No. (%)	2 (3.1)	6 (10.2)	4 (3.6)	12 (5.1)
Total payments per prescriber, median (range), $	4833 (4010-5656)	407 (407-819)	53 208 (32 865-75 000)	2415 (407-75 000)
Food				
Prescribers, No. (%)	51 (78.5)	55 (93.2)	101 (91.0)	207 (88.1)
Total payments per prescriber, median (range), $	118 (12-1184)	273 (11-2068)	206 (12-2773)	183 (11-2773)
Education				
Prescribers, No. (%)	11 (16.9)	16 (27.1)	25 (22.5)	52 (22.1)
Total payments per prescriber, median (range), $	5 (1-18)	5 (1-41)	8 (3-78)	7 (1-78)

In Table 3 and the Figure, we summarize corticotropin use by corticotropin-related payment amount categories. Among the 50 prescribers (21.3%) who received more than $10 000 in payments during the year, corticotropin expenditures per prescriber (mean [SD], $1 304 884 [$1 022 937]) were more than double that of the 45 prescribers (19.2%) who received $25 dollars or less (mean [SD], $594 976 [$256 357]). There were 4 prescribers who received more than $100 000 in payments from Mallinckrodt. There were significant associations between payment amount category and corticotropin expenditures (Kendall τ = 0.29; *P* < .001) and payment amount category and number of prescriptions (Kendall τ = 0.252; *P* < .001) among these frequent prescribers.

**Table 3.** Medicare Repository Corticotropin Use by Corticotropin-Related Payment Level^a^

Total Payments From Mallinckrodt, $	Prescribers, No. (%)	Corticotropin Prescriptions, No.	Corticotropin Prescriptions per Prescriber, Mean (SD), No.	Total Corticotropin Expenditures, $	Corticotropin Expenditures per Prescriber, Mean (SD), $
≤25	45 (19.2)	700	15.6 (5.9)	26 773 924	594 976 (256 357)
26-100	41 (17.5)	644	15.7 (6.0)	26 854 807	654 995 (337 798)
101-1000	80 (34.0)	1458	18.2 (8.5)	61 828 987	772 862 (363 721)
1001-10 000	19 (8.1)	527	27.7 (24.1)	22 553 434	1 187 023 (1 038 448)
>10 000	50 (21.3)	1433	28.7 (21.6)	65 244 183	1 304 884 (1 022 937)

^a^ Significant associations between payment level and mean corticotropin prescriptions (Kendall τ = 0.252; *P* < .001) and between payment level and mean corticotropin expenditures (Kendall τ = 0.29; *P* < .001).

**Figure.**
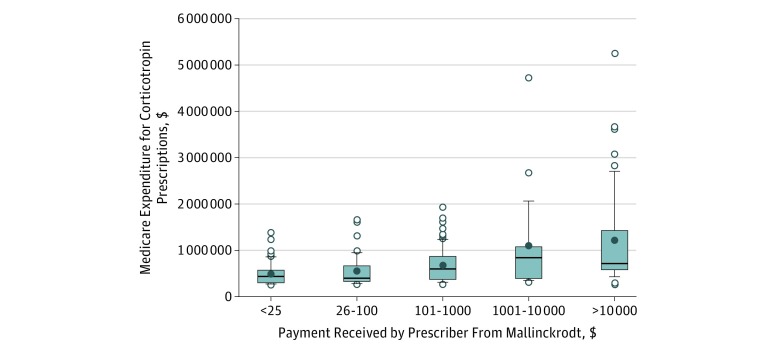
Medicare Spending on Repository Corticotropin by Mallinckrodt Payment Amount The horizontal line in the middle of each box indicates median corticotropin spending per prescriber; bottom and top borders of the box, 25th and 75th percentiles, respectively; whiskers below and above the box, 10th and 90th percentiles; solid circle, mean corticotropin spending per prescriber; and open circles, outliers.

Our multivariable regression analysis is summarized in Table 4 and shows that Medicare spending on corticotropin increased by 7.9% (approximately $53 000) for every $10 000 increase in payments to prescribers (β = 1.079; 95% CI, 1.044-1.115; *P* < .001). The only other significant predictors of Medicare spending on corticotropin were prescriber specialty and sex. In contrast, there was no association between corticotropin-related payments and Medicare spending on corticosteroids (eTable 2 in the Supplement).

**Table 4.** Multivariable Regression Model of Cumulative Repository Corticotropin–Related Payments (Scaled to $10 000) and Log-Transformed Medicare Spending on Corticotropin

Variable	Coefficient (95% CI)	*P* Value
Payment amount^a^	1.0787 (1.0435-1.1152)	<.001
Specialty		
Nephrology	1 [Reference]	<.001
Neurology	0.9600 (0.7721-1.1936)	
Rheumatology	1.4378 (1.2223-1.6913)	
Female	0.8554 (0.7422-0.9858)	.03
Practice size		
1	1 [Reference]	.31
2-10	1.1082 (0.9225-1.3314)	
11-50	0.9220 (0.7438-1.1429)	
>50	1.0645 (0.8871-1.2773)	
Time since graduation, y		
≤10	1 [Reference]	.49
11-30	1.1773 (0.8781-1.5786)	
>30	1.1906 (0.8744-1.6212)	
Not reported	1.4069 (0.9091-2.1772)	
Region		
Northeast	1 [Reference]	.35
Midwest	0.9585 (0.7814-1.1756)	
South	0.9045 (0.762-1.0736)	
Pacific West	1.0532 (0.8356-1.3275)	
Mountain West	0.8003 (0.596-1.0748)	
Total noncorticotropin payments from industry^a^	0.9972 (0.9999-1.0003)	.16
No. of noncorticotropin prescriptions per beneficiary	1.0060 (0.9901-1.0221)	.46
Cost of noncorticotropin prescriptions per claim	1.0001 (0.9934-1.0011)	.40

^a^ Scaled to $10 000.

## Discussion

In our study, 207 of 235 frequent corticotropin prescribers (88%) received a corticotropin-related payment from Mallinckrodt. In 2015, Mallinckrodt made a total of $7 325 957 in general non–research-related corticotropin payments to 10 452 physicians. Of this amount, $1 986 926 (27%) was paid to the 207 frequent corticotropin prescribers (2% of all physicians receiving Mallinckrodt payments for corticotropin) in our study. In contrast, a recent population-based study found that among all specialists, only 35% receive payments from industry.^34^ Although the median total payment for the year was only $189, more than 20% of prescribers received more than $10 000, the top quartile of prescribers received more than $30 000, and several physicians received more than $100 000. The larger dollar amounts given to neurologists were attributable to a higher prevalence of consulting payments or compensation for services other than consulting among these specialists. In general, the category of compensation for services other than consulting had the largest dollar amount per transaction. This category is defined by CMS as “payments made to physicians for speaking, training, and education engagements that are not for continuing education.”^3^ Although precise definitions of reporting categories are not provided, this category likely includes serving on industry speakers’ bureaus and other activities that involve giving talks to physician groups. Finally, we found a significant association between the magnitude of payments and total corticotropin expenditures. Our analysis suggests that every $10 000 spent by Mallinckrodt for payments to physicians is associated with a 7.9% increase in Medicare spending on corticotropin. Based on a median Medicare expenditure per prescriber of $678 706, we would expect a $10 000 increase in payments to yield approximately $53 000 in additional Medicare spending.

Our findings are consistent with other research that examines both the prevalence and extent of influence of pharmaceutical industry payments on physicians. Three large cross-sectional studies that used a similar approach for other medications found that industry payments were positively correlated with increased drug-specific prescribing.^7,8,9^ These studies provide evidence that even small payments, such as meals, are consistently associated with increased prescribing behavior. Additionally, studies focusing on payments by specific manufacturers in several medical specialties report results similar to our own. Two studies evaluating the use of anti–vascular endothelial growth factor injections for eye diseases found payments by Regeneron Pharmaceuticals and Genentech were associated with a higher likelihood of using aflibercept or ranibizumab, respectively, vs lower cost, off-label bevacizumab.^10,35^ Studies of the impact of industry payments on urologists or oncologists treating prostate cancer reveal similar patterns.^6,36,37^

Our findings contrast with the prior literature arising from Open Payments data in several ways. First, about 90% of physicians who frequently prescribed corticotropin received at least 1 payment from Mallinckrodt. Among the ophthalmologists,^10^ urologists,^37^ and oncologists^36^ examined in recent studies of other expensive medications, only 34% to 57% of identified drug prescribers received a manufacturer-related payment. Additionally, payments by Mallinckrodt in the present study appear to be larger than the amounts reported in other studies. In this report, almost a third of frequent prescribers received more than $1000 from Mallinckrodt. In contrast, only 3.8% of prescribers of anti–vascular endothelial growth factor received more than $1000 from the associated manufacturer.^10^ While the reasons for this discrepancy are unclear, it may be attributable to the extent to which Mallinckrodt’s product portfolio depends on corticotropin, which accounted for more than a third of total sales in 2016.^38^ Alternatively, a higher-stakes sales approach may be required to compensate for the lack of compelling evidence justifying corticotropin use over standard synthetic steroids. The most common prescribers of corticotropin are rheumatologists, neurologists, and nephrologists,^27^ suggesting the use of corticotropin in individuals with rheumatic disorders, multiple sclerosis, and nephrotic syndrome. Among these conditions, controlled clinical trial data only support corticotropin use to treat exacerbations of multiple sclerosis,^18,19^ although it is no more effective than methylprednisolone. The use of corticotropin for rheumatoid arthritis,^39^ dermatomyositis and polymyositis,^40,41,42^ systemic lupus erythematosus,^25,43^ and nephrotic syndrome^44,45,46,47,48^ is largely supported by small uncontrolled studies or case series.

### Limitations

Our study has several limitations. First, CMS suppresses prescription information for drugs that have 10 or fewer prescriptions in the Medicare PUF files. Although frequent corticotropin prescribers account for more than half of all corticotropin expenditures by Medicare, it is unclear to what extent our observations generalize to the other 1443 corticotropin prescribers who cannot be identified in the data set. Another issue relates to the accuracy of the Open Payments and Medicare PUF data sets. The Centers for Medicare & Medicaid Services allows and recommends that physicians review payment data and dispute any errors in their records. However, residual inaccuracies may persist. Additionally, Medicare expenditure data do not include any proprietary discounts or rebates, and are therefore likely an overestimate of the net cost to Medicare. Rebates for corticotropin for Medicare are less than federal Medicaid mandatory rebates and are likely around 10%.^32^ Finally, although these data may indicate a causal association between Mallinckrodt payments and corticotropin prescriptions, our study was cross-sectional and the temporal sequence between payments and prescriptions cannot be definitely established. It is conceivable that the company preferentially sought out and supported prominent prescribers of corticotropin.

## Conclusions

Corticotropin is an expensive drug of questionable clinical value that is a large and growing expense for the Medicare program, and perhaps other payers. Our results show that most frequent prescribers of corticotropin in the Medicare program received financial payments from Mallinckrodt, the drug’s maker. Furthermore, we observed a positive association between the amount of money paid to these prescribers, their prescribing intensity, and corticotropin expenditures in the Medicare program with a return on investment for Mallinckrodt of about 5:1. Transparency remains a necessary part of conflict of interest management, and we advocate that physicians who receive significant payments from Mallinckrodt disclose this to patients before prescribing corticotropin for them or consider not receiving any payments from Mallinckrodt. However, disclosure alone may not be sufficient. While academic medical institutions^49^ have implemented conflict of interest policies intended to limit many of the activities observed in this study, those in private practice, like most prescribers in this study, are subject to little oversight. Given the financial consequences for many expensive therapies (both new and old), other stakeholders, including payers, may need to explore other strategies to manage these conflicts.

## Notes

Supplement. **eTable 1.** Non-Repository Corticotropin (ACTH)-Related Payments to Frequent ACTH Prescribers From all Pharmaceutical Companies, Including Mallinckrodt, in 2015 **eTable 2.** Multivariable Regression Model of Cumulative Repository Corticotropin (ACTH)-Related Payments (Scaled to $10,000) and Log-Transformed Medicare Spending on Corticosteroids
