# Supplementary material for: Industry Payments to Physician Specialists Who Prescribe Repository Corticotropin
Source: JAMA Netw Open. 2018 Jun 29;1(2):e180482. doi: 10.1001/jamanetworkopen.2018.0482 (PMC6324413; doi:10.1001/jamanetworkopen.2018.0482)
Supplement: Supplement. — eTable 1. Non-Repository Corticotropin (ACTH)-Related Payments to Frequent ACTH Prescribers From all Pharmaceutical Companies, Including Mallinckrodt, in 2015 eTable 2. Multivariable Regression Model of Cumulative Repository Corticotropin (ACTH)-Related Payments (Scaled to $10,000) and Log-Transformed Medicare Spending on Corticosteroids [file jamanetwopen-1-e180482-s001.pdf]

Supplementary Online Content

Hartung DM, Johnston K, Cohen DM, Nguyen T, Deodhar A, Bourdette DN. Association of industry payments to physician specialists and frequency of repository corticotropin prescription. *JAMA Netw Open*. 2018;1(2):e180482. doi:10.1001/jamanetworkopen.2018.0482

eTable 1. Non-Repository Corticotropin (ACTH)-Related Payments to Frequent ACTH Prescribers From all Pharmaceutical Companies, Including Mallinckrodt, in 2015

eTable 2. Multivariable Regression Model of Cumulative Repository Corticotropin (ACTH)-Related Payments (Scaled to $10,000) and Log-Transformed Medicare Spending on Corticosteroids

This supplementary material has been provided by the authors to give readers additional information about their work.

© 2018 Hartung DM et al. *JAMA Network Open.*

eTable 1: Non-repository corticotropin (ACTH)-related payments to frequent ACTH prescribers from all pharmaceutical companies, including Mallinckrodt, in 2015. Median (minimum and maximum value) reported for those with at least one payment unless noted otherwise

	**Nephrology** (n=65)	**Neurology** (n=59)	**Rheumatology** (n=111)	**Total** (n=235)
Total payments $	$1,154,108	$7,431,598	$11,821,171	$20,406,877
Total transactions	1605	12,932	19,943	34,480
Number of prescribers (%)	60 (92.3)	59 (100)	110 (99.1)	229 (97.5)
Number of transactions per prescriber	12 (1 – 368)	163 (4 – 846)	157 (3 – 875)	94 (1 – 875)
Total payments per prescriber $	$345 ($10 – $449,999)	$22,038 ($130 – $1,428,947)	$7735 ($41 – $1,182,235)	$3988 ($10 – $1,428,947)
Payment per transaction $	$24 ($10 – $9000)	$167 ($11 – $1818)	$79 ($11 – $3021)	$54 ($10 – $9000)
Number of companies per prescriber	6 (1 – 20)	22 (4 – 42)	18 (1 – 42)	14 (1 – 42)

© 2018 Hartung DM et al. *JAMA Network Open.*

eTable 2: Multivariable regression model of cumulative repository corticotropin (ACTH)-related payments (scaled to $10,000) and log-transformed Medicare spending on corticosteroids. Corticosteroids prescriptions include: prednisone, methylprednisolone, prednisolone, dexamethasone, and cortisone.

**Variable**	**Coefficient**	**95% CI**	***P* value**
Payment amount*	1.0637	0.796 to 1.4215	.675
Specialty			<.001
Nephrology (ref)			
Neurology	1.1616	0.1734 to 7.7833	
Rheumatology	640.9170	155.2195 to 2646.4119	
Female	1.0360	0.2999 to 3.5781	.955
Practice size			.812
1 (ref)			
2-10	2.2071	0.4446 to 10.9553	
11-50	1.4807	0.227 to 9.6604	
>50	1.4296	0.291 to 7.0225	
Years since graduation			.023
<=10 (ref)			
11-30	16.9453	1.3084 to 219.4534	
>30	55.9451	3.7756 to 828.9667	
Not reported	12.3133	0.2717 to 558.0285	
Region			.805
Northeast (ref)			
Midwest	2.4030	0.4039 to 14.2959	
South	1.5270	0.3417 to 6.8248	
Pacific west	2.2708	0.3009 to 17.1384	
Mountain west	3.5875	0.2732 to 47.1145	
Total non-ACTH payments from industry*	1.0186	0.9846 to 1.0537	.285
Number of non-ACTH prescriptions per beneficiary	1.2498	1.0878 to 1.436	.002
Cost of non-ACTH prescriptions per claim	0.9963	0.9945 to 0.9981	<.001

*Scaled to $10,000

Missing values of corticosteroid steroid spending were imputed with 0.001 to create log transformation.

© 2018 Hartung DM et al. *JAMA Network Open.*
